# Supplementary material for: Characterizing Methicillin-Resistant Staphylococcus spp. and Extended-Spectrum Cephalosporin-Resistant Escherichia coli in Cattle
Source: Animals (Basel). 2024 Nov 25;14(23):3383. doi: 10.3390/ani14233383 (PMC11640043; doi:10.3390/ani14233383)
Supplement: Supplementary file 1 [file animals-14-03383-s001.zip › animals-3246669 Table S1a_Summary_Pheno-and genotypic characterization of E. coli.pdf]

		Resistance			
Isolate ID	Species	Phenotype**	Genotype	Phylotype****	Virulence associated genes
K32a	*E. coli*	β-lactam, TET, SXT	*$bla_{CTX\text{-}M\text{-}1/15}$, $bla_{TEM\text{-}1}$, tet* (A), *dfrA1* , *sul2* , *aadA1, aadA2*	B1	*fimH1, fimH2*
K59	*E. coli*	β-lactam, SXT	*$bla_{CTX\text{-}M\text{-}1/15}$, $bla_{TEM\text{-}1}$, dfrA14, sul2*	A	*fimH1, fimH2*
K32b	*E. coli*	β-lactam, TET, SXT	*$bla_{CTX\text{-}M1/15}$, aadA1, aadA2, tet(A), sul2, dfrA1*	B1	*fimH1, fimH2,*
K63	*E. coli*	β-lactam, FQR, GEN, TET, SXT, CHL	*$bla_{CTX\text{-}M1/15}$, bla_{TEM} , aadA1, aadA2, aphA, tet(A), sul2, sul3, dfrA12, cmlA1, floR*	A	*fimH1*
K42	*E. coli*	β-lactam, TET, SXT	*$bla_{CTX\text{-}M1/15}$, bla_{TEM} , aadA1, aadA2, tet(A), sul2, dfrA1*	B1	*fimH1, fimH2, papC1, papC2, iucD1, iucD2*
K64	*E. coli*	β-lactam, TET, SXT	*$bla_{CTX\text{-}M1/15}$, aadA4, tet(A), sul2, dfrA17*	B1	*fimH1, fimH2, papC1, papC2, iucD1, iucD2*
K47	*E. coli*	β-lactam, TET, SXT	*$bla_{CTX\text{-}M1/15}$, bla_{TEM} , aadA1, aadA2, tet(A), sul2, dfrA1, dfrA5*	B1	*fimH1, fimH2, papC1, papC2, iucD1, iucD2*
K75	*E. coli*	β-lactam, FQR, TET, SXT, CHL	*$bla_{CTX\text{-}M1/15,}$ bla_{TEM} , aadA4, tet(A), tet(B), dfrA17, cat*	B1	*fimH1, fimH2, papC1, papC2, iucD1, iucD2*
K48	*E. coli*	β-lactam, TET, SXT	*$bla_{CTX\text{-}M1/15}$, bla_{TEM} , aadA1, aadA2, tet(A), sul2, dfrA1, dfrA5*	B1	*fimH1, fimH2, papC1, papC2, iucD1, iucD2*
K89	*E. coli*	β-lactam, SXT	*$blaCTX_{\text{-}M1/15}$, bla_{TEM} , sul2, dfrA14*	A	*fimH1, fimH2*
K50	*E. coli*	β-lactam	*$bla_{CTX\text{-}M9}$, bla_{TEM} , dfrA14*	E clades	*fimH1, fimH2*
K95	*E. coli*	β-lactam, FQR, SXT, CHL	*$bla_{CTX\text{-}M1/15}$, aadA1, aadA2, tet(A), sul3, dfrA12, cmlA1*	B1	*fimH1, hlyA-var2*
K51	*E. coli*	β-lactam, FQR, TET, SXT, CHL	*$bla_{CTX\text{-}M1/15}$, bla_{TEM} , tet(A), sul2, dfrA14, floR*	A	*fimH1, fimH2*
K99	*E. coli*	β-lactam, SXT	*$bla_{CTX\text{-}M1/15}$, bla_{TEM} , sul2, dfrA14*	A	*fimH1, fimH2, papC1, papC2, iucD1, iucD2*
K52	*E. coli*	β-lactam, SXT	*$bla_{CTX\text{-}M1/15}$, bla_{TEM} , sul2, dfrA14*	A	*fimH1, fimH2*
K1	*E. coli*	β-lactam, FQR, GEN, TET, SXT	*$bla_{CTX\text{-}M1/15}$, aadA1, aadA2, aphA, tet(A), sul1, dfrA1, dfrA5*	A	*fimH1, hlyA-var2*
K6	*E. coli*	AmpC, β-lactam, FQR, GEN, TET, SXT,	*$bla_{CTX\text{-}M1/15}$, bla_{TEM} , bla_{ACT} , tet(A), tet(B), sul1, sul2, dfrA5, dfrA7, dfrA17*	B1	*fimH1, fimH2*
K24	*E. coli*	β-lactam, TET, SXT	*$bla_{CTX\text{-}M1/15}$, bla_{TEM} , aadA1, aadA2, tet(A), sul2, dfrA1*	B1	*fimH1, fimH2*
K87	*E. coli*	β-lactam, FQR, TET, SXT, CHL	*$bla_{CTX\text{-}M9}$, bla_{TEM} , dfrA14, floR*	A	*fimH1, fimH2*
K98	*E. coli*	β-lactam, SXT	*$bla_{CTX\text{-}M1/15}$, bla_{TEM} , sul2, dfrA14*	A	*fimH1, fimH2*
K100	*E. coli*	β-lactam, SXT	*$bla_{CTX\text{-}M1/15}$, bla_{TEM} , sul2, dfrA5, dfrA14*	A	*fimH1, fimH2*
K101	*E. coli*	β-lactam, SXT	*$bla_{CTX\text{-}M1/15}$, bla_{TEM} , sul2, dfrA5, dfrA14*	A	*fimH1, fimH2*
