# Supplementary material for: Characterizing Methicillin-Resistant Staphylococcus spp. and Extended-Spectrum Cephalosporin-Resistant Escherichia coli in Cattle
Source: Animals (Basel). 2024 Nov 25;14(23):3383. doi: 10.3390/ani14233383 (PMC11640043; doi:10.3390/ani14233383)
Supplement: Supplementary file 1 [file animals-14-03383-s001.zip › animals-3246669 Table S1b_virulence factors.pdf]

	A	B	C	D	E
1					
2					
3	EXPERIMENT DAT				
4				bundle forming pilu	
5	Sample ID	hp-etx-02	hp-recA-01	hp-tpeL-01	bfpA-10
6	K32a	negative	negative	negative	negative
7	K59	negative	negative	negative	negative
8	K32b	negative	negative	negative	negative
9	K63	negative	negative	negative	negative
10	K63	negative	negative	negative	negative
11	K42	negative	negative	negative	negative
12	K64	negative	negative	negative	negative
13	K47	negative	negative	negative	negative
14	K75	negative	negative	negative	negative
15	K48	negative	negative	negative	negative
16	K89	negative	negative	negative	negative
17	K50	negative	negative	negative	negative
18	K50	negative	negative	negative	negative
19	K95	negative	negative	negative	negative
20	K51	negative	negative	negative	negative
21	K99	negative	negative	negative	negative
22	K52	negative	negative	negative	negative
23	K1	negative	negative	negative	negative
24	K6	negative	negative	negative	negative
25	K24	negative	negative	negative	negative
26	K87	negative	negative	negative	negative
27	K98	negative	negative	negative	negative
28	K100	negative	negative	negative	negative
29	K101	negative	negative	negative	negative

	F	G	H	I	J
1					
2					
3					
4	the attachment to	he attachment to	he attachment to	he attachment to	he attachment to
5	eae - consensus-1	eae - consensus-2	eae - consensus-3	ae - consensus - 4	eae - consensus
6	negative	negative	negative	negative	negative
7	negative	negative	negative	negative	negative
8	negative	negative	negative	negative	negative
9	negative	negative	negative	negative	negative
10	negative	negative	negative	negative	negative
11	negative	negative	negative	negative	negative
12	negative	negative	negative	negative	negative
13	negative	negative	negative	negative	negative
14	negative	negative	negative	negative	negative
15	negative	negative	negative	negative	negative
16	negative	negative	negative	negative	negative
17	negative	negative	negative	negative	negative
18	negative	negative	negative	negative	negative
19	negative	negative	negative	negative	negative
20	negative	negative	negative	negative	negative
21	negative	negative	negative	negative	negative
22	negative	negative	negative	negative	negative
23	negative	negative	negative	negative	negative
24	negative	negative	negative	negative	negative
25	negative	negative	negative	negative	negative
26	negative	negative	negative	negative	negative
27	negative	negative	negative	negative	negative
28	negative	negative	negative	negative	negative
29	negative	negative	negative	negative	negative

	K	L	M	N	O
1					
2					
3	Genes encoding virulence factors - adhesins				
4	brial major subun	brial major subun	fimbrial protein (X	ssociated protein	ssociated protein
5	fasA-10	fedA-10	fim41a-10	hp-acfC01	hp-acfC02
6	negative	negative	negative	negative	negative
7	negative	negative	negative	negative	negative
8	negative	negative	negative	negative	negative
9	negative	negative	negative	negative	negative
10	negative	negative	negative	negative	negative
11	negative	negative	negative	negative	negative
12	negative	negative	negative	negative	negative
13	negative	negative	negative	negative	negative
14	negative	negative	negative	negative	negative
15	negative	negative	negative	negative	negative
16	negative	negative	negative	negative	negative
17	negative	negative	negative	negative	negative
18	negative	negative	negative	negative	negative
19	negative	negative	negative	negative	negative
20	negative	negative	negative	negative	negative
21	negative	negative	negative	negative	negative
22	negative	negative	negative	negative	negative
23	negative	negative	negative	negative	negative
24	negative	negative	negative	negative	negative
25	negative	negative	negative	negative	negative
26	negative	negative	negative	negative	negative
27	negative	negative	negative	negative	negative
28	negative	negative	negative	negative	negative
29	negative	negative	negative	negative	negative

	P	Q	R	S	T
1					
2					
3					
4	for bacterial auto	for bacterial auto	ndle forming pilu	ndle forming pilu	toxic necrotizing f
5	hp-aidA01	hp-aidA02	hp-bfpB01	hp-bfpB02	cnf1-20
6	negative	negative	negative	negative	negative
7	negative	negative	negative	negative	negative
8	negative	negative	negative	negative	negative
9	negative	negative	negative	negative	negative
10	negative	negative	negative	negative	negative
11	negative	negative	negative	negative	negative
12	negative	negative	negative	negative	negative
13	negative	negative	negative	negative	negative
14	negative	negative	negative	negative	negative
15	negative	negative	negative	negative	negative
16	negative	negative	negative	negative	negative
17	negative	negative	negative	negative	negative
18	negative	negative	negative	negative	negative
19	negative	negative	negative	negative	negative
20	negative	negative	negative	negative	negative
21	negative	negative	negative	negative	negative
22	negative	negative	negative	negative	negative
23	negative	negative	negative	negative	negative
24	negative	negative	negative	negative	negative
25	negative	negative	negative	negative	negative
26	negative	negative	negative	negative	negative
27	negative	negative	negative	negative	negative
28	negative	negative	negative	negative	negative
29	negative	negative	negative	negative	negative

	U	V	W	X	Y
1					
2					
3	Genes encoding virulence factors - toxins				
4	ise of enteroaggre	erotoxin subunit A	tabile enterotoxi	t-stabile enteroto	ubunit protein (pi
5	hp-pic-611	ltcA-20	sta1	sta2	f17-G-20
6	negative	negative	negative	negative	negative
7	negative	negative	negative	negative	negative
8	negative	negative	negative	negative	negative
9	negative	negative	negative	negative	negative
10	negative	negative	negative	negative	negative
11	negative	negative	negative	negative	negative
12	negative	negative	negative	negative	negative
13	negative	negative	negative	negative	negative
14	negative	negative	negative	negative	negative
15	negative	negative	negative	negative	negative
16	negative	negative	negative	negative	negative
17	negative	negative	negative	negative	negative
18	negative	negative	negative	negative	negative
19	negative	negative	negative	negative	negative
20	negative	negative	negative	negative	negative
21	negative	negative	negative	negative	negative
22	negative	negative	negative	negative	negative
23	negative	negative	negative	negative	negative
24	negative	negative	negative	negative	negative
25	negative	negative	negative	negative	negative
26	negative	negative	negative	negative	negative
27	negative	negative	negative	negative	negative
28	negative	negative	negative	negative	negative
29	negative	negative	negative	negative	negative

	Z	AA	AB	AC	AD
1					
2					
3	Genes encoding virulence factors - fimbrae				
4	yp I fimbrial prote	yp I fimbrial prote	nbrial protein (fa	nbrial protein (fa	embrane usher P
5	hp-fimH01	hp-fimH02	hp-fimK99-01	hp-fimK99-02	hp-papC01
6	positive	positive	negative	negative	negative
7	positive	positive	negative	negative	negative
8	positive	positive	negative	negative	negative
9	negative	negative	negative	negative	negative
10	positive	negative	negative	negative	negative
11	positive	positive	negative	negative	positive
12	positive	positive	negative	negative	positive
13	positive	positive	negative	negative	positive
14	positive	positive	negative	negative	positive
15	positive	positive	negative	negative	positive
16	positive	positive	negative	negative	negative
17	positive	positive	negative	negative	negative
18	positive	positive	negative	negative	negative
19	positive	negative	negative	negative	negative
20	positive	positive	negative	negative	negative
21	positive	positive	negative	negative	positive
22	positive	positive	negative	negative	negative
23	positive	negative	negative	negative	negative
24	positive	positive	negative	negative	negative
25	positive	positive	negative	negative	negative
26	positive	positive	negative	negative	negative
27	positive	positive	negative	negative	negative
28	positive	positive	negative	negative	negative
29	positive	positive	negative	negative	negative

	AE	AF	AG	AH	AI
1					
2					
3			us- and species-sp	etion system LEE export apparatus	
4	embrane usher P	nit of K88 fimbriae	decarboxylase (A	ction inner memb	ction inner memb
5	hp-papC02	K88ab-10	gad-10	hp-escV01	hp-escV02
6	negative	negative	positive	negative	negative
7	negative	negative	positive	negative	negative
8	negative	negative	positive	negative	negative
9	negative	negative	negative	negative	negative
10	negative	negative	positive	negative	negative
11	positive	negative	positive	negative	negative
12	positive	negative	positive	negative	negative
13	positive	negative	positive	negative	negative
14	positive	negative	positive	negative	negative
15	positive	negative	positive	negative	negative
16	negative	negative	positive	negative	negative
17	negative	negative	positive	negative	negative
18	negative	negative	positive	negative	negative
19	negative	negative	negative	negative	negative
20	negative	negative	positive	negative	negative
21	positive	negative	positive	negative	negative
22	negative	negative	positive	negative	negative
23	negative	negative	negative	negative	negative
24	negative	negative	positive	negative	negative
25	negative	negative	positive	negative	negative
26	negative	negative	positive	negative	negative
27	negative	negative	positive	negative	negative
28	negative	negative	positive	negative	negative
29	negative	negative	positive	negative	negative

	AJ	AK	AL	AM	AN
1					
2					
3	s encoding virulence factors - entero		Genes encoding virulence factors - hamo		
4	ent protein	ent protein	Haemolysin	Haemolysin	Haemolysin
5	hp-espL01	hp-espL02	hp-hlyA-var1-01	hp-hlyA-var1-02	hp-hlyA-var2-01
6	negative	negative	negative	negative	negative
7	negative	negative	negative	negative	negative
8	negative	negative	negative	negative	negative
9	negative	negative	negative	negative	negative
10	negative	negative	negative	negative	negative
11	negative	negative	negative	negative	negative
12	negative	negative	negative	negative	negative
13	negative	negative	negative	negative	negative
14	negative	negative	negative	negative	negative
15	negative	negative	negative	negative	negative
16	negative	negative	negative	negative	negative
17	negative	negative	negative	negative	negative
18	negative	negative	negative	negative	negative
19	negative	negative	negative	negative	negative
20	negative	negative	negative	negative	negative
21	negative	negative	negative	negative	negative
22	negative	negative	negative	negative	negative
23	negative	negative	negative	negative	negative
24	negative	negative	negative	negative	negative
25	negative	negative	negative	negative	negative
26	negative	negative	negative	negative	negative
27	negative	negative	negative	negative	negative
28	negative	negative	negative	negative	negative
29	negative	negative	negative	negative	negative

	AO	AP	AQ	AR	AS
1					
2					
3	lysin	ding virulence factors - aerobactin b		Genes encoding virulence factor	
4	Haemolysin	ctin biosynthesis	ctin biosynthesis	shiga toxin 2	shiga toxin 1
5	hp-hlyA-var2-02	hp-iucD01	hp-iucD02	hp-stxA2-616	stx1
6	negative	negative	negative	negative	negative
7	negative	negative	negative	negative	negative
8	negative	negative	negative	negative	negative
9	negative	negative	negative	negative	negative
10	negative	negative	negative	negative	negative
11	negative	positive	positive	negative	negative
12	negative	positive	positive	negative	negative
13	negative	positive	positive	negative	negative
14	negative	positive	positive	negative	negative
15	negative	positive	positive	negative	negative
16	negative	negative	negative	negative	negative
17	negative	negative	negative	negative	negative
18	negative	negative	negative	negative	negative
19	positive	negative	negative	negative	negative
20	negative	negative	negative	negative	negative
21	negative	positive	positive	negative	negative
22	negative	negative	negative	negative	negative
23	positive	negative	negative	negative	negative
24	negative	negative	negative	negative	negative
25	negative	negative	negative	negative	negative
26	negative	negative	negative	negative	negative
27	negative	negative	negative	negative	negative
28	negative	negative	negative	negative	negative
29	negative	negative	negative	negative	negative

	AT	AU	AV
1			
2			
3	s - shiga toxins		Genes enc
4	shiga toxin 2	enteroinvasive protein	enteroinvasive protein
5	stx2	hp-virB01	hp-virB02
6	negative	negative	negative
7	negative	negative	negative
8	negative	negative	negative
9	negative	negative	negative
10	negative	negative	negative
11	negative	negative	negative
12	negative	negative	negative
13	negative	negative	negative
14	negative	negative	negative
15	negative	negative	negative
16	negative	negative	negative
17	negative	negative	negative
18	negative	negative	negative
19	negative	negative	negative
20	negative	negative	negative
21	negative	negative	negative
22	negative	negative	negative
23	negative	negative	negative
24	negative	negative	negative
25	negative	negative	negative
26	negative	negative	negative
27	negative	negative	negative
28	negative	negative	negative
29	negative	negative	negative

	AW	AX
1		
2		
3	oding virulence factors - invasion	
4	egulator of enteroaggregative E. co	regulator of enteroaggregative E. coli
5	hp-virF-aggR-01	hp-virF-aggR-02
6	negative	negative
7	negative	negative
8	negative	negative
9	negative	negative
10	negative	negative
11	negative	negative
12	negative	negative
13	negative	negative
14	negative	negative
15	negative	negative
16	negative	negative
17	negative	negative
18	negative	negative
19	negative	negative
20	negative	negative
21	negative	negative
22	negative	negative
23	negative	negative
24	negative	negative
25	negative	negative
26	negative	negative
27	negative	negative
28	negative	negative
29	negative	negative
