# Supplementary material for: Characterizing Methicillin-Resistant Staphylococcus spp. and Extended-Spectrum Cephalosporin-Resistant Escherichia coli in Cattle
Source: Animals (Basel). 2024 Nov 25;14(23):3383. doi: 10.3390/ani14233383 (PMC11640043; doi:10.3390/ani14233383)
Supplement: Supplementary file 1 [file animals-14-03383-s001.zip › animals-3246669 Table S1c_genes associated with antibiotic resistance.pdf]

| EXPERIMENT DATA | | Controls | | | Family, genus and species identification | | | | | | | | | | Family-, genus- and species-specific genes | | | | | | | | | | | | | | |
|---|---|---|---|---|---|---|---|---|---|---|---|---|---|---|---|---|---|---|---|---|---|---|---|---|---|---|---|---|---|
| Sample ID | Phylotype | "Failed" indicates problems possibly originating from the staining procedure (see troubleshooting section in the manual). | "Invalid" indicates a contamination of the genomic DNA with ribosomal RNA, which potentially affects the labeling efficiency. | "Failed" indicates possible problems originating from the hybridization or the staining procedure (see troubleshooting section in the manual). | Indicates that the isolate belongs to the species Acinetobacter baumanii. | Indicates that the isolate belongs to the species Citrobacter freundii or Citrobacter braakii. | Indicates that the isolate belongs to the genus Enterobacter. | Summary on family affiliation. "YES" indicates that the isolate belongs to the family Enterobacteriaceae or to the genus Vibrio. "NO" indicates other bacteria. Please note that Proteus spp. may yield "NO". | Indicates that the isolate belongs to the species Escherichia coli. | Indicates that the isolate belongs to the species Klebsiella pneumoniae. | Indicates that the isolate belongs to the species Pseudomonas aeruginosa. | Indicates that the isolate belongs to the genus Salmonella. | Indicates that the isolate belongs to the genus Shigella . | Indicates that the isolate belongs to an enteroinvasive Escherichia coli (EIEC). | glutamate decarboxylase of Escherichia coli (AE014075.1) | integration host factor subunit alpha (U00096.3) | DNA polymerase III subunit alpha (U00096.3) | acinetobactin biosynthesis protein of Acinetobacter baumannii (AY571146.1) | elongation factor P of Acinetobacter baumannii (CP001172.1) | phospholipase D of Acinetobacter baumannii (CP000521.1) | colicin five activity protein of Citrobacter freundii and Citrobacter braakii (U09771.1) | extracytoplasmic function sigma factor of Pseudomonas aeruginosa [LavenirR-JocktaneD-2007] (DQ996558.1) | invasin A, highly specific for genus Salmonella (CP000026.1) | invasion plasmid antigen (AF047365.1) | klebsolysin of Klebsiella pneumoniae (AF293352.1) | lactose permease; the lacY gene is missing in all Shigella spp. (U00096.2) | 16S rRNA (U00096.3) | carbapenemase, class A beta-lactamase (GQ260093.1) | carbapenemase, class B metallo beta-lactamase (KC004136.2) |
| | | gative cont | A contamina | aining contr | bacter baun | ter freundii | terobacter s | robacteriac | scherichia c | iella pneum | monas aeru | lmonella sp | Shigella spp | EIEC | gad | ihfA | dnaE | basC | efp | pld | cfa | ecfX | invA | ipaH9.8 | khe | lacY | rrs | blaBIC | blaDIM |
| K32a | B1 | passed | passed | | NO | NO | NO | YES | YES | NO | NO | NO | NO | NO | positive | positive | positive | negative | negative | negative | negative | negative | negative | negative | negative | positive | positive | negative | negative |
| K59 | A | passed | passed | | NO | NO | NO | YES | YES | NO | NO | NO | NO | NO | positive | positive | positive | negative | negative | negative | negative | negative | negative | negative | negative | positive | positive | negative | negative |
| K32b | B1 | passed | passed | | NO | NO | NO | YES | YES | NO | NO | NO | NO | NO | positive | positive | positive | negative | negative | negative | negative | negative | negative | negative | negative | positive | positive | negative | negative |
| K63 | A | passed | passed | | NO | NO | NO | YES | YES | NO | NO | NO | NO | NO | positive | positive | positive | negative | negative | negative | negative | negative | negative | negative | negative | positive | positive | negative | negative |
| K42 | B1 | passed | passed | | NO | NO | NO | YES | YES | NO | NO | NO | NO | NO | positive | positive | positive | negative | negative | negative | negative | negative | negative | negative | negative | positive | positive | negative | negative |
| K64 | B1 | passed | passed | | NO | NO | NO | YES | YES | NO | NO | NO | NO | NO | positive | positive | positive | negative | negative | negative | negative | negative | negative | negative | negative | positive | positive | negative | negative |
| K47 | B1 | passed | passed | | NO | NO | NO | YES | YES | NO | NO | NO | NO | NO | positive | positive | positive | negative | negative | negative | negative | negative | negative | negative | negative | positive | positive | negative | negative |
| K75 | B1 | passed | passed | | NO | NO | NO | YES | YES | NO | NO | NO | NO | NO | positive | positive | positive | negative | negative | negative | negative | negative | negative | negative | negative | positive | positive | negative | negative |
| K48 | B1 | passed | passed | | NO | NO | NO | YES | YES | NO | NO | NO | NO | NO | positive | positive | positive | negative | negative | negative | negative | negative | negative | negative | negative | positive | positive | negative | negative |
| K89 | A | passed | passed | | NO | NO | NO | YES | YES | NO | NO | NO | NO | NO | positive | positive | positive | negative | negative | negative | negative | negative | negative | negative | negative | positive | positive | negative | negative |
| K50 | E clades | passed | passed | | NO | NO | NO | YES | YES | NO | NO | NO | NO | NO | positive | positive | positive | negative | negative | negative | negative | negative | negative | negative | negative | positive | positive | negative | negative |
| K95 | B1 | passed | passed | | NO | NO | NO | YES | YES | NO | NO | NO | NO | NO | positive | positive | positive | negative | negative | negative | negative | negative | negative | negative | negative | positive | positive | negative | negative |
| K51 | A | passed | passed | | NO | NO | NO | YES | YES | NO | NO | NO | NO | NO | positive | positive | positive | negative | negative | negative | negative | negative | negative | negative | negative | positive | positive | negative | negative |
| K99 | A | passed | passed | | NO | NO | NO | YES | YES | NO | NO | NO | NO | NO | positive | positive | positive | negative | negative | negative | negative | negative | negative | negative | negative | positive | positive | negative | negative |
| K52 | A | passed | passed | | NO | NO | NO | YES | YES | NO | NO | NO | NO | NO | positive | positive | positive | negative | negative | negative | negative | negative | negative | negative | negative | positive | positive | negative | negative |
| K1 | A | passed | passed | | NO | NO | NO | YES | YES | NO | NO | NO | NO | NO | positive | positive | positive | negative | negative | negative | negative | negative | negative | negative | negative | positive | positive | negative | negative |
| K6 | B1 | passed | passed | | NO | NO | NO | YES | YES | NO | NO | NO | NO | NO | positive | positive | positive | negative | negative | negative | negative | negative | negative | negative | negative | positive | positive | negative | negative |
| K20 | - | passed | passed | | NO | YES | NO | YES | YES | NO | NO | NO | NO | NO | positive | positive | positive | negative | negative | negative | positive | negative | negative | negative | negative | positive | positive | negative | negative |
| K24 | B1 | passed | passed | | NO | NO | NO | YES | YES | NO | NO | NO | NO | NO | positive | positive | positive | negative | negative | negative | negative | negative | negative | negative | negative | positive | positive | negative | negative |
| K25 | - | passed | passed | | NO | NO | YES | YES | NO | NO | NO | NO | NO | NO | negative | positive | positive | negative | negative | negative | negative | ambiguous | negative | negative | negative | positive | positive | negative | negative |
| K87 | A | passed | passed | | NO | NO | NO | YES | YES | NO | NO | NO | NO | NO | positive | positive | positive | negative | negative | negative | negative | negative | negative | negative | negative | positive | positive | negative | negative |
| K98 | A | passed | passed | | NO | NO | NO | YES | YES | NO | NO | NO | NO | NO | positive | positive | positive | negative | negative | negative | negative | negative | negative | negative | negative | positive | positive | negative | negative |
| K100 | A | passed | passed | | NO | NO | NO | YES | YES | NO | NO | NO | NO | NO | positive | positive | positive | negative | negative | negative | negative | negative | negative | negative | negative | positive | positive | negative | negative |
| K101 | A | passed | passed | | NO | NO | NO | YES | YES | NO | NO | NO | NO | NO | positive | positive | positive | negative | negative | negative | negative | negative | negative | negative | negative | positive | positive | negative | negative |

| EXPERIMENT DATA | | Carbapenemase genes | | | | | | | | | | | | | | | | | | | | | | | | | | | | | | |
|---|---|---|---|---|---|---|---|---|---|---|---|---|---|---|---|---|---|---|---|---|---|---|---|---|---|---|---|---|---|---|---|---|
| Sample ID | Phylotype | carbapenemase, class A beta-lactamase (AY219651.1)<br>blaGES | carbapenemase, class B metallo beta-lactamase (consensus)<br>IM (conse | carbapenemase, class B metallo beta-lactamase (consensus))<br>OB (conse | carbapenemase, class A beta-lactamase associated with impenem resistance (AY780889.1)<br>IMI-3 (Nm | regulator of blaIMI-3 (NMC-A) (Z21956.1)<br>blaIMI-R | carbapenemase, class B metallo beta-lactamase (consensus)<br>MP (conse | carbapenemase, class B metallo beta-lactamase (synonym: blaSIM) (EU686387.1)<br>IP25 (blaS | carbapenemase, class B metallo beta-lactamase (JQ432564.1)<br>blaIMP35 | carbapenemase, class B metallo beta-lactamase of Chryseobacterium (consensus)<br>ND (conse | carbapenemase, class B metallo beta-lactamase (consensus))<br>HM (conse | carbepenemase, class A serin beta-lactamase (consensus)<br>PC (conse | carbapenemase, class B metallo beta-lactamase (New Dehli metallo beta-lactamase) (consensus)<br>DM (conse | carbapenemase, subclass B3 metallo beta-lactamase (Pseudomonas alcaligenes metallo-betalactamase) (AB858498.1)<br>blaPAM-1 | carbapenemase, class B metallo beta-lactamase (AF197943.1)<br>blaSFH-1 | carbapenemase, class B metallo beta-lactamase (Serratia marcescens) (AB636283.1)<br>blaSMB-1 | carbapenemase, class A beta-lactamase (Serratia marcescens) (consensus)<br>consensus | carbapenemase, class B metallo beta-lactamase (AY341249.1)<br>blaSPM-1 | carbapenemase, class B metallo beta-lactamase (consensus)<br>IM (conse | carbapenemase, class B metallo beta-lactamase (AF191564.1)<br>blaVIM-2 | carbapenemase, class B metallo beta-lactamase (AJ536835.1)<br>blaVIM-7 | carbapenemase, class D beta-lactamase (AJ132105.1) | carbapenemase, class D beta-lactamase (AF509241.1)<br>blaOXA-40-li | carbapenemase, class D beta-lactamase (AY236073.2)<br>OXA-48-li | carbapenemase, class D beta-lactamase (CP000863.1)<br>OXA-51-li | Insertion sequence ABa1 is adjacent to blaOXA-51-like gene. This combination mediated carbapenem resistant in Acinetobacter baumannii isolates (Turton et al. 2006). (CP001921.1)<br>1 to blaO | Insertion sequence ABa1 is not adjacent to blaOXA-51-like gene. This combination does not mediate carbapenem resistant in Acinetobacter baumannii isolates (Turton et al. 2006). (CU459141.1)<br>Ba1 to bla | carbapenemase, class D beta-lactamase (AY500137.1)<br>blaOXA-54 | carbapenemase, class D beta-lactamase (AY343493.1)<br>blaOXA-55 | carbapenemase, class D beta-lactamase (AY665723.1)<br>blaOXA-58 | carbapenemase blaOXA-134 or -235 or -284, class D beta-lactamase (AYHO01000005.1)<br>XA-134/23 | carbapenemase blaOXA-40-like, class D beta-lactamase (GQ861437.1)<br>-143/182/ |
| K32a | B1 | negative | negative | negative | negative | negative | negative | negative | negative | negative | negative | negative | negative | negative | negative | negative | negative | negative | negative | negative | negative | negative | negative | negative | negative | negative | negative | negative | negative | negative | negative | negative |
| K59 | A | negative | negative | negative | negative | negative | negative | negative | negative | negative | negative | negative | negative | negative | negative | negative | negative | negative | negative | negative | negative | negative | negative | negative | negative | negative | negative | negative | negative | negative | negative | negative |
| K32b | B1 | negative | negative | negative | negative | negative | negative | negative | negative | negative | negative | negative | negative | negative | negative | negative | negative | negative | negative | negative | negative | negative | negative | negative | negative | negative | negative | negative | negative | negative | negative | negative |
| K63 | A | negative | negative | negative | negative | negative | negative | negative | negative | negative | negative | negative | negative | negative | negative | negative | negative | negative | negative | negative | negative | negative | negative | negative | negative | negative | negative | negative | negative | negative | negative | negative |
| K42 | B1 | negative | negative | negative | negative | negative | negative | negative | negative | negative | negative | negative | negative | negative | negative | negative | negative | negative | negative | negative | negative | negative | negative | negative | negative | negative | negative | negative | negative | negative | negative | negative |
| K64 | B1 | negative | negative | negative | negative | negative | negative | negative | negative | negative | negative | negative | negative | negative | negative | negative | negative | negative | negative | negative | negative | negative | negative | negative | negative | negative | negative | negative | negative | negative | negative | negative |
| K47 | B1 | negative | negative | negative | negative | negative | negative | negative | negative | negative | negative | negative | negative | negative | negative | negative | negative | negative | negative | negative | negative | negative | negative | negative | negative | negative | negative | negative | negative | negative | negative | negative |
| K75 | B1 | negative | negative | negative | negative | negative | negative | negative | negative | negative | negative | negative | negative | negative | negative | negative | negative | negative | negative | negative | negative | negative | negative | negative | negative | negative | negative | negative | negative | negative | negative | negative |
| K48 | B1 | negative | negative | negative | negative | negative | negative | negative | negative | negative | negative | negative | negative | negative | negative | negative | negative | negative | negative | negative | negative | negative | negative | negative | negative | negative | negative | negative | negative | negative | negative | negative |
| K89 | A | negative | negative | negative | negative | negative | negative | negative | negative | negative | negative | negative | negative | negative | negative | negative | negative | negative | negative | negative | negative | negative | negative | negative | negative | negative | negative | negative | negative | negative | negative | negative |
| K50 | E clades | negative | negative | negative | negative | negative | negative | negative | negative | negative | negative | negative | negative | negative | negative | negative | negative | negative | negative | negative | negative | negative | negative | negative | negative | negative | negative | negative | negative | negative | negative | negative |
| K95 | B1 | negative | negative | negative | negative | negative | negative | negative | negative | negative | negative | negative | negative | negative | negative | negative | negative | negative | negative | negative | negative | negative | negative | negative | negative | negative | negative | negative | negative | negative | negative | negative |
| K51 | A | negative | negative | negative | negative | negative | negative | negative | negative | negative | negative | negative | negative | negative | negative | negative | negative | negative | negative | negative | negative | negative | negative | negative | negative | negative | negative | negative | negative | negative | negative | negative |
| K99 | A | negative | negative | negative | negative | negative | negative | negative | negative | negative | negative | negative | negative | negative | negative | negative | negative | negative | negative | negative | negative | negative | negative | negative | negative | negative | negative | negative | negative | negative | negative | negative |
| K52 | A | negative | negative | negative | negative | negative | negative | negative | negative | negative | negative | negative | negative | negative | negative | negative | negative | negative | negative | negative | negative | negative | negative | negative | negative | negative | negative | negative | negative | negative | negative | negative |
| K1 | A | negative | negative | negative | negative | negative | negative | negative | negative | negative | negative | negative | negative | negative | negative | negative | negative | negative | negative | negative | negative | negative | negative | negative | negative | negative | negative | negative | negative | negative | negative | negative |
| K6 | B1 | negative | negative | negative | negative | negative | negative | negative | negative | ambiguous | negative | negative | negative | negative | negative | negative | negative | negative | negative | negative | negative | negative | negative | negative | negative | negative | negative | negative | negative | negative | negative | negative |
| K20 | - | negative | negative | negative | negative | negative | negative | negative | negative | negative | negative | negative | negative | negative | negative | negative | negative | negative | negative | negative | negative | negative | negative | negative | negative | negative | negative | negative | negative | negative | negative | negative |
| K24 | B1 | negative | negative | negative | negative | negative | negative | negative | negative | negative | negative | negative | negative | negative | negative | negative | negative | negative | negative | negative | negative | negative | negative | negative | negative | negative | negative | negative | negative | negative | negative | negative |
| K25 | - | negative | negative | negative | negative | negative | negative | negative | negative | negative | negative | negative | negative | negative | negative | negative | negative | negative | negative | negative | negative | negative | negative | negative | negative | negative | negative | negative | negative | negative | negative | negative |
| K87 | A | negative | negative | negative | negative | negative | negative | negative | negative | negative | negative | negative | negative | negative | negative | negative | negative | negative | negative | negative | negative | negative | negative | negative | negative | negative | negative | negative | negative | negative | negative | negative |
| K98 | A | negative | negative | negative | negative | negative | negative | negative | negative | negative | negative | negative | negative | negative | negative | negative | negative | negative | negative | negative | negative | negative | negative | negative | negative | negative | negative | negative | negative | negative | negative | negative |
| K100 | A | negative | negative | negative | negative | negative | negative | negative | negative | negative | negative | negative | negative | negative | negative | negative | negative | negative | negative | negative | negative | negative | negative | negative | negative | negative | negative | negative | negative | negative | negative | negative |
| K101 | A | negative | negative | negative | negative | negative | negative | negative | negative | negative | negative | negative | negative | negative | negative | negative | negative | negative | negative | negative | negative | negative | negative | negative | negative | negative | negative | negative | negative | negative | negative | negative |

| EXPERIMENT DATA | | | | | | Extended-spectrum beta-lactamase genes | | | | | | | | | | | | | Other beta-lactamase genes | | | | | AmpC genes | | | | | | | | |
|---|---|---|---|---|---|---|---|---|---|---|---|---|---|---|---|---|---|---|---|---|---|---|---|---|---|---|---|---|---|---|---|---|
| Sample ID | Phylotype | carbapenemase blaOXA-48-like, class D beta-lactamase (CP000469.1) | carbapenemase, class D beta-lactamase (JN861783.1) | carbapenemase, class D beta-lactamase (APOK01000044.1) | carbapenemase, class D beta-lactamase (APRH01000012.1) | extended spectrum beta-lactamase, class A (AF033200.1) | extended spectrum beta-lactamase, class A (X92506.1), including blaCTX-M15 (HQ202266.1) | extended spectrum beta-lactamase, class A (AF286192.1) | extended spectrum beta-lactamase, class A (AY750914.2) | extended spectrum beta-lactamase, class A (FQ482074.1) | extended-spectrum beta-lactamase precursor, class C (AF381617.1) | extended-spectrum beta-lactamase, class A beta-lactamase PER-1 (Z21957.1) | extended-spectrum beta-lactamase, class A beta-lactamase PER-2 (X93314.1) | class A beta-lactamase - consensus sequence for blaSHV genes, including extended-spectrum beta-lactamases | class A beta-lactamase - consensus sequence for blaTEM genes, including extended-spectrum beta-lactamases | extended-spectrum beta-lactamase, class A (consensus) | extended spectrum beta-lactamase, class D (EU503121.1) | extended spectrum beta-lactamase, class D (AJ519683.1) | narrow spectrum beta-lactamase, class D (AY458016.1) | narrow spectrum beta-lactamase, class D (M55547.1) | consensus probe for extended and narrow spectrum class D beta-lactamases belonging to group blaOXA-2 | consensus probe for extended and narrow spectrum class D beta-lactamases belonging to group blaOXA-10 | narrow spectrum beta-lactamase, class D (AF525303.2) | extended spectrum beta-lactamase, class C beta-lactamase (M37839.2) | AmpC beta-lactamase (EF554600.1) | AmpC beta-lactamase (U58495.2) | AmpC beta-lactamase, consensus sequence for blaCMY-13, blaCMY-2, blaCMY-24, blaCMY-35, blaCMY-CFE1, blaCMY-CFE2 (Citrobacter spp.), blaCMY-Cmur (Citrobacter murliniae), blaCMY-Cwer (Citrobacter werkmanii), blaCMY-Cyou (Citrobacte [illegible] | AmpC beta-lactamase (EF406115.1) | AmpC beta-lactamase (consensus) | AmpC beta-lactamase (consensus) | 3-N-aminoglycoside acetyltransferase; associated with resistance to astromicin; gentamicin; sisomicin (consensus) | 3-N-aminoglycoside acetyltransferase; associated with resistance to astromicin; gentamicin; sisomicin (U90945.1) |
| | | OXA-181/ | blaOXA-21 | blaOXA-27 | blaOXA-29 | blaCME | aCTX-M1/ | blaCTX-M2 | blaCTX-M8 | blaCTX-M9 | aMOX-CM | blaPER-1 | blaPER-2 | HV (conse | EM (conse | EB (conse | blaOXA-18 | blaOXA-45 | blaOXA-1 | blaOXA-9 | A-2 (cons | A-10 (cons | blaOXA-60 | blaMIR | blaACC | blaACT | MY (conse | blaDHA | OX (conse | OX (conse | 3') (conser | aac(3')-Ia |
| K32a | B1 | negative | negative | negative | negative | negative | positive | negative | negative | negative | negative | negative | negative | negative | positive | negative | negative | negative | negative | negative | negative | negative | negative | negative | negative | negative | negative | negative | negative | negative | negative | negative |
| K59 | A | negative | negative | negative | negative | negative | positive | negative | negative | negative | negative | negative | negative | negative | positive | negative | negative | negative | negative | negative | negative | negative | negative | negative | negative | negative | negative | negative | negative | negative | negative | negative |
| K32b | B1 | negative | negative | negative | negative | negative | positive | negative | negative | negative | negative | negative | negative | negative | negative | negative | negative | negative | negative | negative | negative | negative | negative | negative | negative | negative | negative | negative | negative | negative | negative | negative |
| K63 | A | negative | negative | negative | negative | negative | positive | negative | negative | negative | negative | negative | negative | negative | positive | negative | negative | negative | negative | negative | negative | negative | negative | negative | negative | negative | negative | negative | negative | negative | negative | negative |
| K42 | B1 | negative | negative | negative | negative | negative | positive | negative | negative | negative | negative | negative | negative | negative | positive | negative | negative | negative | ambiguous | negative | negative | negative | negative | negative | negative | negative | negative | negative | negative | negative | negative | negative |
| K64 | B1 | negative | negative | negative | negative | negative | positive | negative | negative | negative | negative | negative | negative | negative | negative | negative | negative | negative | negative | negative | negative | negative | negative | negative | negative | negative | negative | negative | negative | negative | negative | negative |
| K47 | B1 | negative | negative | negative | negative | negative | positive | negative | negative | negative | negative | negative | negative | negative | positive | negative | negative | negative | negative | negative | negative | negative | negative | negative | negative | negative | negative | negative | negative | negative | negative | negative |
| K75 | B1 | negative | negative | negative | negative | negative | positive | negative | negative | negative | negative | negative | negative | negative | positive | negative | negative | negative | negative | negative | negative | negative | negative | negative | negative | negative | negative | negative | negative | negative | negative | negative |
| K48 | B1 | negative | negative | negative | negative | negative | positive | negative | negative | negative | negative | negative | negative | negative | positive | negative | negative | negative | negative | negative | negative | negative | negative | negative | negative | negative | negative | negative | negative | negative | negative | negative |
| K89 | A | negative | negative | negative | negative | negative | positive | negative | negative | negative | negative | negative | negative | negative | positive | negative | negative | negative | negative | negative | negative | negative | negative | negative | negative | negative | negative | negative | negative | negative | negative | negative |
| K50 | E clades | negative | negative | negative | negative | negative | negative | negative | negative | positive | negative | negative | negative | negative | positive | negative | negative | negative | negative | negative | negative | negative | negative | negative | negative | negative | negative | negative | negative | negative | negative | negative |
| K95 | B1 | negative | negative | negative | negative | negative | positive | negative | negative | negative | negative | negative | negative | negative | negative | negative | negative | negative | negative | negative | negative | negative | negative | negative | negative | negative | negative | negative | negative | negative | negative | negative |
| K51 | A | negative | negative | negative | negative | negative | positive | negative | negative | negative | negative | negative | negative | negative | positive | negative | negative | negative | negative | negative | negative | negative | negative | negative | negative | negative | negative | negative | negative | negative | negative | negative |
| K99 | A | negative | negative | negative | negative | negative | positive | negative | negative | negative | negative | negative | negative | negative | positive | negative | negative | negative | negative | negative | negative | negative | negative | negative | negative | negative | negative | negative | negative | negative | negative | negative |
| K52 | A | negative | negative | negative | negative | negative | positive | negative | negative | negative | negative | negative | negative | negative | positive | negative | negative | negative | ambiguous | negative | negative | negative | negative | negative | negative | negative | negative | negative | negative | negative | negative | negative |
| K1 | A | negative | negative | negative | negative | negative | positive | negative | negative | negative | negative | negative | negative | negative | negative | negative | negative | negative | negative | negative | negative | negative | negative | negative | negative | ambiguous | negative | negative | negative | negative | negative | negative |
| K6 | B1 | negative | negative | negative | negative | negative | positive | negative | negative | negative | negative | negative | negative | negative | positive | negative | negative | negative | negative | negative | negative | negative | negative | negative | negative | positive | negative | negative | negative | negative | negative | negative |
| K20 | - | negative | negative | negative | negative | negative | negative | negative | negative | negative | ambiguous | negative | negative | negative | negative | negative | ambiguous | negative | negative | negative | negative | negative | negative | negative | negative | positive | positive | negative | negative | negative | negative | negative |
| K24 | B1 | negative | negative | negative | negative | negative | positive | negative | negative | negative | negative | negative | negative | negative | positive | negative | negative | negative | negative | negative | negative | negative | negative | negative | negative | negative | negative | negative | negative | negative | negative | negative |
| K25 | - | negative | negative | negative | negative | negative | negative | negative | negative | negative | negative | negative | negative | negative | negative | negative | negative | negative | negative | negative | negative | negative | negative | positive | negative | positive | negative | negative | negative | negative | negative | negative |
| K87 | A | negative | negative | negative | negative | negative | negative | negative | negative | positive | negative | negative | negative | negative | positive | negative | negative | negative | negative | negative | negative | negative | negative | negative | negative | negative | negative | negative | negative | negative | negative | negative |
| K98 | A | negative | negative | negative | negative | negative | positive | negative | negative | negative | negative | negative | negative | negative | positive | negative | negative | negative | negative | negative | negative | negative | negative | negative | negative | ambiguous | negative | negative | negative | negative | negative | negative |
| K100 | A | negative | negative | negative | negative | negative | positive | negative | negative | negative | negative | negative | negative | negative | positive | negative | negative | negative | negative | negative | negative | negative | negative | negative | negative | ambiguous | negative | negative | negative | negative | negative | negative |
| K101 | A | negative | negative | negative | negative | negative | positive | negative | negative | negative | negative | negative | negative | negative | positive | negative | negative | negative | negative | negative | negative | negative | negative | negative | negative | ambiguous | negative | negative | negative | negative | negative | negative |

| EXPERIMENT DATA | | Genes associated with aminoglycoside resistance | | | | | | | | | | | | | | | | | | | | | | | | Genes associated with macrolide resistance | | Genes as | |
|---|---|---|---|---|---|---|---|---|---|---|---|---|---|---|---|---|---|---|---|---|---|---|---|---|---|---|---|---|---|
| Sample ID | Phylotype | 3-N-aminoglycoside acetyltransferase; associated with resistance to astromicin; gentamicin; sisomicin (KJ679408.1) | 3-N-aminoglycoside acetyltransferase; associated with resistance to astromicin; gentamicin; sisomicin (AJ511268.1) | 3-N-aminoglycoside acetyltransferase; associated with resistance to astromicin; gentamicin; sisomicin (AY458224.1) | 3-N-aminoglycoside acetyltransferase; associated with resistance to apramycin; dibekacin; gentamicin; netilmicin; sisomicin; tobramycin (EU784152.1) | aminoglycoside 6'-N-acetyltransferase, associated with resistance to amikacin; dibekacin; isepamicin; netilmicin; sisomicin; tobramycin (consensus) | aminoglycoside 6'-N-acetyltransferase; associated with resistance to streptomycin, spectinomycin (AJ640197.1) | aminoglycoside 6'-N-acetyltransferase; associated with resistance to streptomycin, spectinomycin (M21682.1) | aminoglycoside 6'-N-acetyltransferase; associated with resistance to streptomycin, spectinomycin (EF127959.1) | aminoglycoside 6'-N-acetyltransferase; associated with resistance to streptomycin, spectinomycin (EU912537.1) | aminoglycoside 6'-N-acetyltransferase; associated with resistance to streptomycin, spectinomycin (EU855788.1) | 6'-aminoglycoside-N-acetyltransferase/2''-aminoglycoside phosphotransferase; associated with resistance to gentamycin (AE017171.1) | aminoglycoside adenyltransferase; associated with resistance to streptomycin, spectinomycin (EU704128.1) | aminoglycoside adenyltransferase; associated with resistance to streptomycin, spectinomycin (EU704128.1) | aminoglycoside adenyltransferase; associated with resistance to streptomycin, spectinomycin (Z50802.3) | 2''-aminoglycoside nucleotidyltransferase (L06418.4) | aminoglycoside (2'') adenylyltransferase; associated with resistance to dibekacin; gentamicin; kanamycin; sisomicin; tobramycin (L06418.4) | aminoglycoside 3'-phosphotransferase; kanamycin resistance protein (AY260546.3) | 16S rRNA methylase, associated with aminoglycoside resistance (AB117519.1) | 16S rRNA methylase, associated with gentamicin resistance (M55521.1) | 16S rRNA methylase, associated with aminoglycoside resistance (AB261016.1) | 16S rRNA methylase, associated with aminoglycoside resistance (AB083212.2) | 16S rRNA methylase, associated with aminoglycoside resistance (DQ345788.1) | 16S rRNA methylase, associated with aminoglycoside resistance (AB194779.2) | 16S rRNA methylase, associated with aminoglycoside resistance (DQ914960.2) | macrolide 2'-phosphotransferase (consensus) | member of macrolide inactivation gene cluster mphA-mrx-mphR (consensus) | QepA - fluoroquinolone/quinolone efflux pump (AM886293.1) | quinolone or fluoroquinolone resistance protein (AY931018.1) |
| | | aac(3')-Ib | aac(3')-Ic | aac(3')-Ie | aac(3')-IVa | 6') (conser | aac(6')-31 | aac(6')-Ib | aac(6')-II | aac(6')-IIa | aac(6')-IIc | aac-aph | aadA1 | aadA2 | aadA4 | aadB | ant2 | aphA | armA | grm | npmA | rmtA | rmtB | rmtC | rmtD | h (consens | mrx (consensus) | qepA | qnrA1 |
| K32a | B1 | negative | negative | negative | negative | negative | negative | negative | negative | negative | negative | negative | positive | positive | negative | negative | negative | negative | negative | negative | negative | negative | negative | negative | negative | positive | positive | negative | negative |
| K59 | A | negative | negative | negative | negative | negative | negative | negative | negative | negative | negative | negative | negative | negative | negative | negative | negative | negative | negative | negative | negative | negative | negative | negative | negative | negative | negative | negative | negative |
| K32b | B1 | negative | negative | negative | negative | negative | negative | negative | negative | negative | negative | negative | positive | positive | negative | negative | negative | negative | negative | negative | negative | negative | negative | negative | negative | positive | positive | negative | negative |
| K63 | A | negative | negative | negative | negative | negative | negative | negative | negative | negative | negative | negative | positive | positive | negative | negative | negative | positive | negative | negative | negative | negative | negative | negative | negative | negative | negative | negative | negative |
| K42 | B1 | negative | negative | negative | negative | negative | negative | negative | negative | negative | negative | negative | positive | positive | negative | negative | negative | negative | negative | negative | negative | negative | negative | negative | negative | positive | positive | negative | negative |
| K64 | B1 | negative | negative | negative | negative | negative | negative | negative | negative | negative | negative | negative | negative | negative | positive | negative | negative | negative | negative | negative | negative | negative | negative | negative | negative | negative | negative | negative | negative |
| K47 | B1 | negative | negative | negative | negative | negative | negative | negative | negative | negative | negative | negative | positive | positive | negative | negative | negative | negative | negative | negative | negative | negative | negative | negative | negative | positive | positive | negative | negative |
| K75 | B1 | negative | negative | negative | negative | negative | negative | negative | negative | negative | negative | negative | negative | negative | positive | negative | negative | negative | negative | negative | negative | negative | negative | negative | negative | negative | negative | negative | negative |
| K48 | B1 | negative | negative | negative | negative | negative | negative | negative | negative | negative | negative | negative | positive | positive | negative | negative | negative | negative | negative | negative | negative | negative | negative | negative | negative | positive | positive | negative | negative |
| K89 | A | negative | negative | negative | negative | negative | negative | negative | negative | negative | negative | negative | negative | negative | negative | negative | negative | negative | negative | negative | negative | negative | negative | negative | negative | negative | negative | negative | negative |
| K50 | E clades | negative | negative | negative | negative | negative | negative | negative | negative | negative | negative | negative | negative | negative | negative | negative | negative | negative | negative | negative | negative | negative | negative | negative | negative | negative | negative | negative | negative |
| K95 | B1 | negative | negative | negative | negative | negative | negative | negative | negative | negative | negative | negative | positive | positive | negative | negative | negative | negative | negative | negative | negative | negative | negative | negative | negative | positive | positive | negative | negative |
| K51 | A | negative | negative | negative | negative | negative | negative | negative | negative | negative | negative | negative | negative | negative | negative | negative | negative | negative | negative | negative | negative | negative | negative | negative | negative | negative | negative | negative | negative |
| K99 | A | negative | negative | negative | negative | negative | negative | negative | negative | negative | negative | negative | negative | negative | negative | negative | negative | negative | negative | negative | negative | negative | negative | negative | negative | negative | negative | negative | negative |
| K52 | A | negative | negative | negative | negative | negative | negative | negative | negative | negative | negative | negative | negative | negative | negative | negative | negative | negative | negative | negative | negative | negative | negative | negative | negative | negative | negative | negative | negative |
| K1 | A | negative | negative | negative | negative | negative | negative | negative | negative | negative | negative | negative | positive | positive | negative | negative | negative | positive | negative | negative | negative | negative | negative | negative | negative | positive | positive | negative | negative |
| K6 | B1 | negative | negative | negative | negative | negative | negative | negative | negative | negative | negative | negative | negative | negative | negative | negative | negative | negative | negative | negative | negative | negative | negative | negative | negative | ambiguous | negative | negative | negative |
| K20 | - | negative | negative | negative | negative | negative | negative | negative | negative | negative | negative | negative | negative | negative | negative | ambiguous | negative | negative | negative | negative | negative | negative | negative | negative | negative | ambiguous | negative | negative | negative |
| K24 | B1 | negative | negative | negative | negative | negative | negative | negative | negative | negative | negative | negative | positive | positive | negative | negative | negative | negative | negative | negative | negative | negative | negative | negative | negative | positive | positive | negative | negative |
| K25 | - | negative | negative | negative | negative | negative | negative | negative | negative | negative | negative | negative | ambiguous | positive | negative | negative | negative | negative | negative | negative | negative | negative | negative | negative | negative | ambiguous | negative | negative | negative |
| K87 | A | negative | negative | negative | negative | negative | negative | negative | negative | negative | negative | negative | negative | negative | negative | negative | negative | negative | negative | negative | negative | negative | negative | negative | negative | negative | negative | negative | negative |
| K98 | A | negative | negative | negative | negative | negative | negative | negative | negative | negative | negative | negative | negative | negative | negative | negative | negative | negative | negative | negative | negative | negative | negative | negative | negative | negative | negative | negative | negative |
| K100 | A | negative | negative | negative | negative | negative | negative | negative | negative | negative | negative | negative | negative | negative | negative | negative | negative | negative | negative | negative | negative | negative | negative | negative | negative | ambiguous | negative | negative | negative |
| K101 | A | negative | negative | negative | negative | negative | negative | negative | negative | negative | negative | negative | negative | negative | negative | negative | negative | negative | negative | negative | negative | negative | negative | negative | negative | positive | negative | negative | negative |

| EXPERIMENT DATA | | sociated with quinolone resistance | | | | Genes associated with sulphonamide resistance | | | Genes associated with trimethoprim resistance | | | | | | | | | Genes encoding virulence factors - miscellaneous | | | | Genes associated with a multidrug efflux pump | | Toxin-antitoxin system | | |
|---|---|---|---|---|---|---|---|---|---|---|---|---|---|---|---|---|---|---|---|---|---|---|---|---|---|---|
| Sample ID | Phylotype | quinolone or fluoroquinolone resistance protein (AB281054.1) | quinolone or fluoroquinolone resistance protein (EU917444.1) | quinolone or fluoroquinolone resistance protein (FJ228229.1) | quinolone or fluoroquinolone resistance protein (AM234722.1) | dihydropteroate synthetase type 1 (AJ698325.1) | dihydropteroate synthetase type 2 (DQ464881.1) | dihydropteroate synthetase type 3 (AJ459418.2) | dihydrofolate reductase type 1 (AJ884723.1) | dihydrofolate reductase type 5 (AB188269.1) | dihydrofolate reductase type 7 (AB161450.1, AM237806.1) | dihydrofolate reductase type 12 (AB154407.1) | dihydrofolate reductase type 13 (synonym A21) (Z50802.3) | dihydrofolate reductase type 14 (AJ313522.1) | dihydrofolate reductase type 15 (Z83311.1) | dihydrofolate reductase type 17 (AF169041.1) | dihydrofolate reductase type 19 (AJ310778.1) | class 1 integron integrase (AY260546.3) | class 2 integron integrase (AY183453.1) | class 3 integron integrase (EF469602.1) | transposase for the transposon ISEcp1 (AB543698.1) | OqxA - membran fusion protein, component of RND-type multidrug efflux pump, associated with olaquindox resistance (EU370913.1) | OqxB - integral membrane protein, component of RND-type multidrug efflux pump, associated with olaquindox resistance (EU370913.1) | higA is the antitoxin of the translation-dependent mRNA interferase toxin higB (U43847.1) | Ectopic expression of higB causes inhibition of cell growth which is alleviated by co-expression of higA (U43847.1) | splA is the antitoxin of the translation-dependent mRNA interferase toxin splT (EU294228.1) |
| | | qnrB | qnrC | qnrD | qnrS | sul1 | sul2 | sul3 | dfrA1 | dfrA5 | dfrA7 | dfrA12 | dfrA13 | dfrA14 | dfrA15 | dfrA17 | dfrA19 | intI1 | intI2 | intI3 | tnpISEcp1 | oqxA | oqxB | higA | higB | splA |
| K32a | B1 | negative | negative | negative | negative | negative | positive | negative | positive | negative | negative | negative | negative | negative | negative | negative | negative | positive | negative | negative | negative | negative | negative | negative | negative | negative |
| K59 | A | negative | negative | negative | positive | negative | positive | negative | negative | negative | negative | negative | negative | positive | negative | negative | negative | negative | negative | negative | positive | negative | negative | negative | negative | negative |
| K32b | B1 | negative | negative | negative | negative | negative | positive | negative | positive | negative | negative | negative | negative | negative | negative | negative | negative | positive | negative | negative | negative | negative | negative | negative | negative | negative |
| K63 | A | negative | negative | negative | negative | negative | positive | positive | negative | ambiguous | negative | positive | negative | negative | negative | negative | negative | positive | negative | negative | negative | negative | negative | negative | negative | negative |
| K42 | B1 | negative | negative | negative | negative | negative | positive | negative | positive | negative | negative | negative | negative | negative | negative | negative | negative | positive | negative | negative | negative | negative | negative | negative | negative | negative |
| K64 | B1 | negative | negative | negative | negative | negative | positive | negative | negative | negative | negative | negative | negative | negative | negative | positive | negative | positive | negative | negative | positive | negative | negative | negative | negative | negative |
| K47 | B1 | negative | negative | negative | negative | negative | positive | negative | positive | positive | negative | negative | negative | negative | negative | negative | negative | positive | negative | negative | negative | negative | negative | negative | negative | negative |
| K75 | B1 | negative | negative | negative | negative | negative | negative | negative | negative | negative | negative | negative | negative | negative | negative | positive | negative | positive | negative | negative | positive | negative | negative | negative | negative | negative |
| K48 | B1 | negative | negative | negative | negative | negative | positive | negative | positive | positive | negative | negative | negative | negative | negative | negative | negative | positive | negative | negative | negative | negative | negative | negative | negative | negative |
| K89 | A | negative | negative | negative | positive | negative | positive | negative | negative | negative | negative | negative | negative | positive | negative | negative | negative | negative | negative | negative | positive | negative | negative | negative | negative | negative |
| K50 | E clades | negative | negative | negative | positive | negative | negative | negative | negative | negative | negative | negative | negative | positive | negative | negative | negative | positive | negative | negative | negative | negative | negative | negative | negative | negative |
| K95 | B1 | negative | negative | negative | negative | negative | negative | positive | negative | negative | negative | positive | negative | negative | negative | negative | negative | positive | negative | negative | negative | negative | negative | negative | negative | negative |
| K51 | A | negative | negative | negative | positive | negative | positive | negative | negative | negative | negative | negative | negative | positive | negative | negative | negative | negative | negative | negative | positive | negative | negative | negative | negative | negative |
| K99 | A | negative | negative | negative | positive | negative | positive | negative | negative | negative | negative | negative | negative | positive | negative | negative | negative | negative | negative | negative | positive | negative | negative | negative | negative | negative |
| K52 | A | negative | negative | negative | positive | negative | positive | negative | negative | negative | negative | negative | negative | positive | ambiguous | negative | negative | negative | negative | negative | positive | negative | negative | negative | negative | negative |
| K1 | A | negative | negative | negative | positive | positive | negative | negative | positive | positive | negative | negative | negative | negative | negative | negative | negative | positive | negative | negative | negative | negative | negative | negative | negative | negative |
| K6 | B1 | negative | negative | negative | negative | positive | positive | negative | negative | positive | positive | negative | negative | negative | negative | positive | negative | positive | negative | negative | negative | negative | negative | negative | negative | negative |
| K20 | - | negative | negative | negative | negative | negative | ambiguous | negative | negative | positive | negative | negative | negative | negative | negative | negative | negative | negative | negative | negative | negative | negative | negative | negative | negative | negative |
| K24 | B1 | negative | negative | negative | negative | negative | positive | negative | positive | negative | negative | negative | negative | negative | negative | negative | negative | positive | negative | negative | negative | negative | negative | negative | negative | negative |
| K25 | - | negative | negative | negative | negative | positive | negative | negative | negative | positive | negative | negative | negative | negative | negative | negative | negative | positive | negative | negative | negative | positive | positive | negative | negative | negative |
| K87 | A | negative | negative | negative | positive | negative | negative | negative | negative | ambiguous | negative | negative | negative | positive | negative | negative | negative | positive | negative | negative | negative | negative | negative | negative | negative | negative |
| K98 | A | negative | negative | negative | positive | negative | positive | negative | negative | ambiguous | negative | negative | negative | positive | negative | negative | negative | negative | negative | negative | positive | negative | negative | negative | negative | negative |
| K100 | A | negative | negative | negative | positive | negative | positive | negative | negative | positive | negative | negative | negative | positive | negative | negative | negative | negative | negative | negative | positive | negative | negative | negative | negative | negative |
| K101 | A | negative | negative | negative | positive | negative | positive | negative | negative | positive | negative | negative | negative | positive | negative | negative | negative | negative | negative | negative | positive | negative | negative | negative | negative | negative |

| EXPERIMENT DATA | | | Genes associated with colistin resistance | | | | | | | | |
|---|---|---|---|---|---|---|---|---|---|---|---|
| Sample ID | Phylotype | Ectopic expression of splT causes inhibition of cell growth which is alleviated by co-expression of splA (EU294228.1) | consensus probe for genes associated with colistin resistance (mcr-1 to mcr-9 ) | mcr-1 and mcr-2, phosphoethanolamine transferase associated with resistance to colistin and polymyxin-type antibiotics (KP347127.1) | mcr-3, phosphoethanolamine transferase associated with resistance to colistin and polymyxin-type antibiotics (KY924928.1) | mcr-4, phosphoethanolamine transferase associated with resistance to colistin and polymyxin-type antibiotics (MF543359.1.1) | mcr-5, phosphoethanolamine transferase associated with resistance to colistin and polymyxin-type antibiotics (NG_055658.1) | mcr-6, phosphoethanolamine transferase associated with resistance to colistin and polymyxin-type antibiotics (NG_055781.1) | mcr-7, phosphoethanolamine transferase associated with resistance to colistin and polymyxin-type antibiotics (NG_056413.1) | mcr-8, phosphoethanolamine transferase associated with resistance to colistin and polymyxin-type antibiotics (NG_061399.1) | mcr-9, phosphoethanolamine transferase associated with resistance to colistin and polymyxin-type antibiotics (MK070339.1) |
| | | splT | cr_consens | cr-1 / mcr | mcr-3 | mcr-4 | mcr-5 | mcr-6 | mcr-7 | mcr-8 | mcr-9 |
| K32a | B1 | negative | negative | negative | negative | negative | negative | negative | negative | negative | negative |
| K59 | A | negative | negative | negative | negative | negative | negative | negative | negative | negative | negative |
| K32b | B1 | negative | negative | negative | negative | negative | negative | negative | negative | negative | negative |
| K63 | A | negative | negative | negative | negative | negative | negative | negative | negative | negative | negative |
| K42 | B1 | negative | negative | negative | negative | negative | negative | negative | negative | negative | negative |
| K64 | B1 | negative | negative | negative | negative | negative | negative | negative | negative | negative | negative |
| K47 | B1 | negative | negative | negative | negative | negative | negative | negative | negative | negative | negative |
| K75 | B1 | negative | negative | negative | negative | negative | negative | negative | negative | negative | negative |
| K48 | B1 | negative | negative | negative | negative | negative | negative | negative | negative | negative | negative |
| K89 | A | negative | negative | negative | negative | negative | negative | negative | negative | negative | negative |
| K50 | E clades | negative | negative | negative | negative | negative | negative | negative | negative | negative | negative |
| K95 | B1 | negative | negative | negative | negative | negative | negative | negative | negative | negative | negative |
| K51 | A | negative | negative | negative | negative | negative | negative | negative | negative | negative | negative |
| K99 | A | negative | negative | negative | negative | negative | negative | negative | negative | negative | negative |
| K52 | A | negative | negative | negative | negative | negative | negative | negative | negative | negative | negative |
| K1 | A | negative | negative | negative | negative | negative | negative | negative | negative | negative | negative |
| K6 | B1 | negative | negative | negative | negative | negative | negative | negative | negative | negative | negative |
| K20 | - | negative | negative | negative | negative | negative | negative | negative | negative | negative | negative |
| K24 | B1 | negative | negative | negative | negative | negative | negative | negative | negative | negative | negative |
| K25 | - | negative | negative | negative | negative | negative | negative | negative | negative | negative | negative |
| K87 | A | negative | negative | negative | negative | negative | negative | negative | negative | negative | negative |
| K98 | A | negative | negative | negative | negative | negative | negative | negative | negative | negative | negative |
| K100 | A | negative | negative | negative | negative | negative | negative | negative | negative | negative | negative |
| K101 | A | negative | negative | negative | negative | negative | negative | negative | negative | negative | negative |
