# Supplementary material for: Characterizing Methicillin-Resistant Staphylococcus spp. and Extended-Spectrum Cephalosporin-Resistant Escherichia coli in Cattle
Source: Animals (Basel). 2024 Nov 25;14(23):3383. doi: 10.3390/ani14233383 (PMC11640043; doi:10.3390/ani14233383)
Supplement: Supplementary file 1 [file animals-14-03383-s001.zip › animals-3246669 Table S2_Target Genes.pdf]

TARGET GENES

Species Markers

mD1	domain 1 of 23S-rRNA
gapA	glyceraldehyde 3-phosphate dehydrogenase, locus 1
katA	katalase A
coA	Coagulase
nuc1	thermostable extracellular nuclease
spa	staphylococcal protein A
sbi	IgG binding protein

Regulatory Genes

sarA	staphylococcal accessory regulator A
saeS	histidine protein kinase, sae locus
vraS	sensor protein
agrI (total)	accesory gene regulator allele I
agrB-I	accesory gene regulator allele I
agrC-I	accesory gene regulator allele I
agrD-I	accesory gene regulator allele I
agrII (total)	accesory gene regulator allele II
agrB-II	accesory gene regulator allele II
agrC-II	accesory gene regulator allele II
agrD-II	accesory gene regulator allele II
agrIII (total)	accesory gene regulator allele III
agrB-III	accesory gene regulator allele III
agrC-III	accesory gene regulator allele III
agrD-III	accesory gene regulator allele III
agrIV (total)	accesory gene regulator allele IV
agrB-IV	accesory gene regulator allele IV
agrC-IV	accesory gene regulator allele IV
hld	haemolysin delta

Resistance Genes

Methicillin Resistance and SCC*mec* Typing

mecA	alternate penicillin binding protein 2, defining MRSA
mecC	novel *mecA* homologue, also associated with beta lactam resistance
delta_mecR	truncated signal transducer protein MecR1

ugpQ	glycerophosphoryl diester phosphodiesterase, associated with *mecA*
ccrA-1	cassette chromosome recombinase gene, A-1
ccrB-1	cassette chromosome recombinase gene, B-1
plsSCC (COL)	plasmin-sensitive surface protein
Q9XB68-dcs	hypothetical protein from SCC*mec* elements
ccrA-2	cassette chromosome recombinase gene, A-2
ccrB-2	cassette chromosome recombinase gene, B-2
kdpA-SCC	potassium translocating ATPase A, chain 2
kdpB-SCC	potassium translocating ATPase B, chain 1
kdpC-SCC	potassium translocating ATPase C, chain 2
kdpD-SCC	sensor kinase protein
kdpE-SCC	KDP operon transcriptional regulatory protein
meci	methicillin-resistance gene regulatory protein
mecR	signal transducer protein *Mec*R1
xylR	homolog of xylose repressor, associated with SCC*mec* elements
ccrA-3	cassette chromose recombinase gene, A-3
ccrB-3	cassette chromose recombinase gene, B-3
merA	mercury resistance gene operon, Hg(II) reductase
merB	mercury resistance gene operon, alcylmercury lyase
ccrAA (MRSAZH47)_probe1	Putative protein, homologue to cassette chromosome recombinase A genes
ccrAA (MRSAZH47)_probe2	
ccrC (85-2082)	cassette chromosome recombinase gene C
ccrA-4	cassette chromosome recombinase gene A-4
ccrB-4	cassette chromosome recombinase gene B-4
**Penicillinase**	
blaZ	beta-lactamase gene
blaZ-SCCmec XI	beta-lactamase gene associated with SCC*mec* XI elements
blaI	beta-lactamase repressor (inhibitor)
blaR	beta-lactamase regulatory protein
**MLS-Antibiotics**	
erm(A)	rRNA methyltransferase associated with macrolide/lincosamide resistance
erm(B)	rRNA methyltransferase associated with macrolide/lincosamide resistance
erm(C)	rRNA methyltransferase associated with macrolide/lincosamide resistance
Inu(A)	lincosamide nucleotidyltransferase (=linA)
msr(A)	macrolide efflux pump
mef(A)	macrolide efflux protein A
mph(C)	macrolide phosphotransferase II (=mpbBM)
vat(A)	virginiamycin A acetyltransferase
vat(B)	acetyltransferase inactivating streptogramin A

vga(A)	ABC transporter conferring resistance to streptogramin A and related compounds
vga(A) (BM3327)	*vga(A)* allele from strain BM 3327
vga(B)	virginiamycin B hydrolase (=vgb)
**Aminoglycosides**	
aacA-aphD	aminoglycoside adenyl-/phosphotransferase (genatmycin, tobramycin)
aadD	aminoglycoside adenyltransferase (neo-/kanamycin, tobramycin)
aphA3	aminoglycoside phosphotransferase (neo-/kanamycin)
**Miscellaneous Genes**	
sat	streptothricin acetyltransferase
dfrS1	dihydrofolate reductase mediating trimethoprim resistance (=dfrA)
fusB	fusidic acid resistance gene (=far1)
fusC	fusidic acid resistance gene (=Q6GD50)
mupA	isoleucyl-tRNA synthetase associated with mupirocin resistance (=mupR)
tet(K)	tetracycline efflux protein
tet(M)	ribosomal protection protein associated with tetracycline resistance
cat(total)	chloramphenicol acetyltransferase
cat (pC221)	chloramphenicol acetyltransferase
cat(pc223)	chloramphenicol acetyltransferase
cat(pMC524)	chloramphenicol acetyltransferase
cat(pSBK203R)	chloramphenicol acetyltransferase
cfr	23S-rRNA methyltransferase (phenicols, lincosamides, oxazolidinones, pleuromutilins, streptogramin A)
fexA	chloramphenicol/florfenicol exporter
fosB	metallothiol transferase
fosB (plasmid)	metallothiol transferase
**Efflux Systems**	
qacA	quaternary ammonium compound/multidrug efflux protein C
qacC (total)	quaternary ammonium compound/multidrug efflux protein A
qacC (equine)	quaternary ammonium compound/multidrug efflux protein A
qacC (consensus)	quaternary ammonium compound/multidrug efflux protein A
qacC (SA5)	quaternary ammonium compound/multidrug efflux protein A
qacC (Ssap)	quaternary ammonium compound/multidrug efflux protein A

qacC (ST94)	quaternary ammonium compound/multidrug efflux protein A
sdrM	putative transport protein (=tetEfflux)
**Glycopeptides**	
vanA	vancomycin resistance gene
vanB	vancomycin resistance gene from enterococci and *Clostridium*
vanZ	teicoplanin resistance gene from enterococci

Virulence Markers

**Toxic Shock Syndrome Toxin**	
tst1 (consensus)	toxic shock syndrom toxin 1
tsts1 ("human" allele)	toxic shock syndrom toxin 2
tsts1 ("bovine" allele, from RF122)	toxic shock syndrom toxin 3
**Enterotoxins**	
sea	enterotoxin A (=*entA*)
sea (320E)	enterotoxin A, allele from strain 320E
sea (N315)	enterotoxin A, allele from strain N315 = enterotoxin P
seb	enterotoxin B (=*entB*)
sec	enterotoxin C (=*entC*)
sed	enterotoxin d (=*entD*)
see	enterotoxin E (=*entE*)
seg	enterotoxin G (=*entG*)
seh	enterotoxin H (=*entH*)
sei	enterotoxin I (=*entI*)
sej	enterotoxin J (=*entJ*)
sek	enterotoxin K (=*entK*)
sel	enterotoxin L (=*entL*)
selm	enterotoxin-like gene/protein M (=*sem, entM*)
seln (consensus)	enterotoxin-like gene/protein N (=*seN, entN*)
seln (other than RF122)	enterotoxin-like gene/protein N (=*seN, entN*)
selo	enterotoxin-like gene/protein O (=*seo, entO*)
egc	enterotoxin gene cluster (seg/i/selm/n/o/u)
seq	enterotoxin Q (=*entQ*)
ser	enterotoxin R (=*entR*)
selu	enterotoxin-like gene/protein U (=*seu, entU*)
ORF CM14_probe1	enterotoxin-like protein ORF CM14
ORF CM14_probe2	enterotoxin-like protein ORF CM15
**HLG and Leukocidins**	
lukF	haemolysin gamma/leukocidin, component B (F)
lukS	haemolysin gamma/leukocidin, component C (S)
lukS (ST22, ST45)	haemolysin gamma/leukocidin, component C (S), allele from ST22 and ST45
hlgA	haemolysin gamma, component A
lukF-PV	Panton Valentine leukocidin F component
lukS-PV	Panton Valentine leukocidin S component

lukF-PV (P83)	F component of leukocidin from ruminants
lukM	S component of leukocidin from ruminants
lukD	leukocidin D component
lukE	leukocidin E component
lukX	leukocidin/haemolysin toxin family protein
lukY	leukocidin/haemolysin toxin family protein
lukY (ST30, ST45)	leukocidin/haemolysin toxin family protein, allele from ST30 and ST45
**Haemolysins**	
hl	putative membrane protein
hla	haemolysin alpha
hIIII (consensus)	putative membrane protein
hIIII (other than RF122)	putative membrane protein
hlb_probe1	haemolysin beta
hlb_probe2	haemolysin beta
hlb_probe3	haemolysin beta
un-disrupted hlb	haemolysin beta without phage insertion
**HLB-Converting Phages**	
sak	Staphylokinase
chp	chemotaxis-inhibiting protein (CHIPS)
scn	staphylococcal complement inhibitor
**Exfoliative Toxins**	
etA	exfoliative toxin protein serotype A
etB	exfoliative toxin protein serotype B
etD	exfoliative toxin D
**Epidermal Cell Differentation Inhibitor (EDIN)**	
edinA	epidermal cell differentation inhibitor
edinB	epidermal cell differentation inhibitor B
edinC	epidermal cell differentation inhibitor C
**ACME Locus**	
ACME cluster	Arginine Catabolic Mobile Element
arcA-SCC	ACME-locus: arginine deiminase
arcB-SCC	ACME-locus: ornithincarbamoyltransferase
arcC-SCC	ACME-locus: carbamatkinase
arcD-SCC	ACME-locus: arginine/ornithine-antiporter
**Proteases**	
aur (consensus)	Aureolysin
aur (other than MRSA252)	Aureolysin
aur (MRSA 252)	Aureolysin
splA	serinprotease A
splB	serinprotease B
splE	serinprotease E
sspA	Glutamylendopeptidase
sspB	staphopain B, protease
sspP (consensus)	staphopain A (staphylopain A), protease
sspP (other than ST93)	staphopain A (staphylopain A), protease
**Staphylococcal Superantigen/Enterotoxin-like Genes (SET/SSL)**	

setC/selx	staphylococcal exotoxin-like protein/Sag gene homolog, SAUSA300_0370
ssl01/set6_probe1_11	staphylococcal superantigen-like protein 1 (probes)
ssl01/set6_probe2_11	staphylococcal superantigen-like protein 1 (probes)
ssl01/set6_probe1_12	staphylococcal superantigen-like protein 1 (probes)
ssl01/set6_probe2_12	staphylococcal superantigen-like protein 1 (probes)
ssl01/set6_probe4_11	staphylococcal superantigen-like protein 1 (probes)
ssl01/set6_probeRF122	staphylococcal superantigen-like protein 1 (probes)
ssl01/set6 (COL)	staphylococcal superantigen-like protein 1 (interpretation/alleles)
ssl01/set6 (Mu50, N315)	staphylococcal superantigen-like protein 1 (interpretation/alleles)
ssl01/set6 (MW2, MSSA476)	staphylococcal superantigen-like protein 1 (interpretation/alleles)
ssl01/set6 (MRSA252)	staphylococcal superantigen-like protein 1 (interpretation/alleles)
ssl01/set6 (RF122)	staphylococcal superantigen-like protein 1 (interpretation/alleles)
ssl01/set6 (other alleles)	staphylococcal superantigen-like protein 1 (interpretation/alleles)
ssl02/set7	staphylococcal superantigen-like protein 2
ssl02/set7 (MRSA252)	staphylococcal superantigen-like protein 2
ssl03/set8_probe1	staphylococcal superantigen-like protein 3
ssl03/set8_probe2	staphylococcal superantigen-like protein 3
ssl03/set8 (MRSA252, SAR0424)	staphylococcal superantigen-like protein 3
ssl04/set9	staphylococcal superantigen-like protein 4
ssl04/set9 (MRSA252, SAR0424)	staphylococcal superantigen-like protein 4
ssl05/set3_probe1	staphylococcal superantigen-like protein 5
ssl05/set3 (RF122, probe-611)	staphylococcal superantigen-like protein 5
ssl05/set3_probe2 (612)	staphylococcal superantigen-like protein 5
ssl05/set3 (MRSA252)	staphylococcal superantigen-like protein 5
ssl06/set21	staphylococcal superantigen-like protein 6
ssl06 (NCTC8325, MW2)	staphylococcal superantigen-like protein 6
ssl07/set1	staphylococcal superantigen-like protein 7
ssl07/set1 (MRSA252)	staphylococcal superantigen-like protein 7
ssl07/set1 (AF188836)	staphylococcal superantigen-like protein 7
ssl08/set12_probe1	staphylococcal superantigen-like protein 8
ssl08/set12_probe2	staphylococcal superantigen-like protein 8
ssl09/set5_probe1	staphylococcal superantigen-like protein 9
ssl09/set5_probe2	staphylococcal superantigen-like protein 9
ssl09/set5 (MRSA252)	staphylococcal superantigen-like protein 9

ssl10/set4	staphylococcal superantigen-like protein 10
ssl10 (RF122)	staphylococcal superantigen-like protein 10
ssl10/set4 (MRSA252)	staphylococcal superantigen-like protein 10
ssl11/set2 (COL)	staphylococcal superantigen-like protein 11
ssl11+set2 (Mu50, N315)	staphylococcal superantigen-like protein 11
ssl11+set2 (MW2, MSSA476)	staphylococcal superantigen-like protein 11
ssl11/set2 (MRSA252)	staphylococcal superantigen-like protein 11
setB3	staphylococcal exotoxin-like protein, second locus
setB3 (MRSA252)	staphylococcal exotoxin-like protein, second locus
setB2	staphylococcal exotoxin-like protein, second locus
setB2 (MRSA252)	staphylococcal exotoxin-like protein, second locus
setB1	staphylococcal exotoxin-like protein, second locus

Miscellaneous Typing Genes

Capsule- and Biofilm-Associated Genes

cap1 (total)	capsule type 1
capH1	capsular polysaccharide synthesis enzyme
capJ1	O-antigen polymerase
capK1	capsular polysaccharide biosynthesis protein
cap5 (total)	capsule type 5
capH5	capsular polysaccharide synthesis enzyme
capJ5	O-antigen polymerase
capK5	capsular polysaccharide biosynthesis protein
cap 8 (total)	capsule type 8
capH8	capsular polysaccharide synthesis enzyme
capI8	capsular polysaccharide biosynthesis protein
capJ8	O-antigen polymerase
capK8	capsular polysaccharide biosynthesis protein
icaA	intercellular adhesion protein A
icaC	intercellular adhesion protein C
icaD	intercellular adhesion protein D
Bap	surface protein involved in biofilm formation

Adhesion Factors/Genes Encoding Microbial Surface Components Recognizin Adhesive Matrix Molecules (Mscramm Genes)

bbp (total)	bone sialoprotein-binding protein
bbp (consensus)	bone sialoprotein-binding protein
bbp (COL, MW2)	bone sialoprotein-binding protein
bbp (MRSA252)	bone sialoprotein-binding protein
bbp (Mu50)	bone sialoprotein-binding protein
bbp (RF122)	bone sialoprotein-binding protein
bbp (ST45)	bone sialoprotein-binding protein
clfA (total)	clumping factor A
clfA (consensus)	clumping factor A
clfa (COL, RF122)	clumping factor A
clfA (MRSA252)	clumping factor A
clfA (Mu50, MW2)	clumping factor A
clfB (total)	clumping factor B

clfB (consensus)	clumping factor B
clfB (COL, Mu50)	clumping factor B
clfB (MW2)	clumping factor B
clfB (RF122)	clumping factor B
Cna	collagen-binding adhesin
ebh (consensus)	cell wall associated fibronectin-binding protein
ebpS (total)	cell surface elastin binding protein
ebpS_probe612	cell surface elastin binding protein
ebpS_probe614	cell surface elastin binding protein
ebpS (01-1111)	cell surface elastin binding protein
ebpS (COL)	cell surface elastin binding protein
Eno	Enolase
fib	fibrinogen binding protein (19kDa)
fib (MRSA252)	fibrinogen binding protein (19kDa)
fnbA (total)	fibronectin-binding protein A
fnbA (consensus)	fibronectin-binding protein A
fnbA (COL)	fibronectin-binding protein A
fnbA (MRSA252)	fibronectin-binding protein A
fnbA (Mu50, MW2)	fibronectin-binding protein A
fnbA (RF122)	fibronectin-binding protein A
fnbB (total)	fibronectin-binding protein B
fnbB (COL)	fibronectin-binding protein B
fnbB (COL, Mu50, MW2)	fibronectin-binding protein B
fnbB (Mu50)	fibronectin-binding protein B
fnbB (MW2)	fibronectin-binding protein B
fnbB (ST15)	fibronectin-binding protein B
fnbB (ST45-2)	fibronectin-binding protein B
map (total)	major histocompatibility complex class II analog protein (=Extracellular adherence protein, eap)
map (COL)	major histocompatibility complex class II analog protein (=Extracellular adherence protein, eap)
map (MRSA252)	major histocompatibility complex class II analog protein (=Extracellular adherence protein, eap)
map (Mu50, MW2)	major histocompatibility complex class II analog protein (=Extracellular adherence protein, eap)
sasG (total)	Staphylococcus aureus surface protein G
sasG (COL, Mu50)	Staphylococcus aureus surface protein G
sasG (MW2)	Staphylococcus aureus surface protein G
sasG (other than MRSA252, RF122)	Staphylococcus aureus surface protein G
sdrC (total)	Ser-Asp rich fibrinogen-/bone sialoprotein-binding protein C
sdrC (consensus)	Ser-Asp rich fibrinogen-/bone sialoprotein-binding protein C
sdrC (B1)	Ser-Asp rich fibrinogen-/bone sialoprotein-binding protein C
sdrC (COL)	Ser-Asp rich fibrinogen-/bone sialoprotein-binding protein C

sdrC (Mu50)	Ser-Asp rich fibrinogen-/bone sialoprotein-binding protein C
sdrC (MW2, MRSA252, RF122)	Ser-Asp rich fibrinogen-/bone sialoprotein-binding protein C
sdrC (other than MRSA252, RF122)	Ser-Asp rich fibrinogen-/bone sialoprotein-binding protein C
sdrD (total)	Ser-Asp rich fibrinogen-/bone sialoprotein-binding protein D
sdrD (consensus)	Ser-Asp rich fibrinogen-/bone sialoprotein-binding protein D
sdrD (COL, MW2)	Ser-Asp rich fibrinogen-/bone sialoprotein-binding protein D
sdrD (Mu50)	Ser-Asp rich fibrinogen-/bone sialoprotein-binding protein D
sdrD (other)	Ser-Asp rich fibrinogen-/bone sialoprotein-binding protein D
vwb (total)	van Willebrand factor binding protein
vwb (consensus)	van Willebrand factor binding protein
vwb (COL, MW2)	van Willebrand factor binding protein
vw (MRSA252)	van Willebrand factor binding protein
vwb (Mu50)	van Willebrand factor binding protein
vwb (RF122)	van Willebrand factor binding protein
**Immunodominant Antigen B**	
isaB	immunodominant antigen B
isaB (MRSA252)	immunodominant antigen B
**Defensin Resistance**	
mprF (COL, MW2)	defensin resistance gene protein
mprF (Mu50, MRSA252)	defensin resistance gene protein
**Transferrin Binding Protein**	
isdA (consensus)	transferrin-binding protein
isdA (MRSA252)	transferrin-binding protein
isdA (other than MRSA252)	transferrin-binding protein
**Putative Transporter**	
ImrP (other than RF122)_probe 1	hypothetical protein, similar to integral membrane protein LmrP
ImrP (other than RF122)_probe 2	hypothetical protein, similar to integral membrane protein LmrP
ImrP (RF122)_probe1	hypothetical protein, similar to integral membrane protein LmrP
ImrP (RF122)_probe2	hypothetical protein, similar to integral membrane protein LmrP
**Type I Restriction-Modification System, Single Sequence Specificity Protein**	
hsdS1 (RF122)	type I site-specific deoxyribonuclease subunit, 1st locus
hsdS2 (Mu50, N315, COL, USA300, NCTC8325)	type I site-specific deoxyribonuclease subunit, 2nd locus

hsdS2 (MW2, MSSA476)	type I site-specific deoxyribonuclease subunit, 2nd locus
hsdS2(RF122)	type I site-specific deoxyribonuclease subunit, 2nd locus
hsdS2 (MRSA252)	type I site-specific deoxyribonuclease subunit, 2nd locus
hsdS3 (all other than RF122, MRSA252)	type I site-specific deoxyribonuclease subunit, 3rd locus
hsdS3 (COL, USA300, NCTC8325, MW2, MSSA476, RF122)	type I site-specific deoxyribonuclease subunit, 3rd locus
hsdS3 (Mu50, N315)	type I site-specific deoxyribonuclease subunit, 3rd locus
hsdS3 (CC51, MRSA252)	type I site-specific deoxyribonuclease subunit, 3rd locus
hsdS3 (MRSA252)	type I site-specific deoxyribonuclease subunit, 3rd locus
hsdSx (CC25)	type I site-specific deoxyribonuclease subunit, unknown locus
hsdSx (CC15)	type I site-specific deoxyribonuclease subunit, unknown locus
hsdSx (etd)	type I site-specific deoxyribonuclease subunit, unknown locus
**Miscellaneous Genes**	
Q2FXC0	hypothetical protein, located next to serine protease operon
Q2YUB3	unspecific efflux/transporter
Q7A4X2	hypothetical protein
**Hyaluronate Lyase**	
hysA1 (MRSA252)	hyaluronate lyase, first/second locus
hysA1 (MRSA252, RF122) and/or hysA2(consensus)	hyaluronate lyase, first/second locus
hysA1 (MRSA252, RF122) and/or hysA2(COL, USA300)	hyaluronate lyase, first/second locus
hysA2 (all other than MRSA252)	hyaluronate lyase/second locus
hysA2 (COL, USA300, NCTC8325)	hyaluronate lyase/second locus
hysA2 (all other than COL, USA300, NCTC8325)_probe1	hyaluronate lyase/second locus
hysA2 (all other than COL, USA300, NCTC8325)_probe2	hyaluronate lyase/second locus
hysA2 (MRSA252)	hyaluronate lyase/second locus
