# Supplementary figures and images for: Characterizing Methicillin-Resistant Staphylococcus spp. and Extended-Spectrum Cephalosporin-Resistant Escherichia coli in Cattle
Source: Animals (Basel). 2024 Nov 25;14(23):3383. doi: 10.3390/ani14233383 (PMC11640043; doi:10.3390/ani14233383)

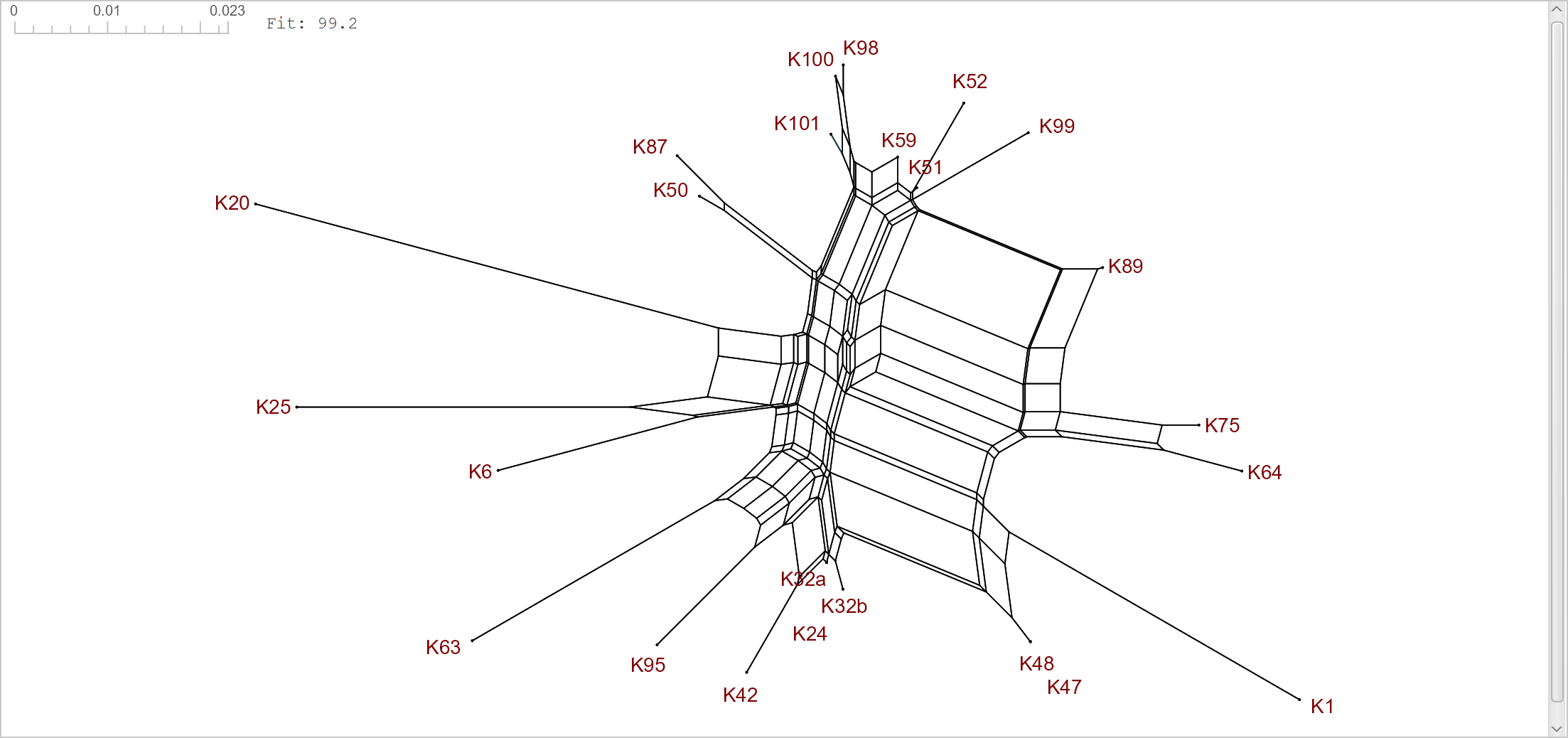

Supplement: Supplementary file 1 [file animals-14-03383-s001.zip › FigureS1_Splitstree.png]
